# Calcium insensitivity of FA-6, a cell line derived from a pancreatic cancer associated with humoral hypercalcemia, is mediated by the significantly reduced expression of the Calcium Sensitive Receptor transduction component p38 MAPK

**DOI:** 10.1186/1476-4598-5-51

**Published:** 2006-11-01

**Authors:** Richard Morgan, Benjamin Fairfax, Hardev S Pandha

**Affiliations:** 1 Postgraduate Medical School, University of Surrey, UK

## Abstract

The Calcium-Sensing Receptor is a key component of Calcium/Parathyroid hormone homeostatic system that helps maintain appropriate plasma Ca^2+ ^concentrations. It also has a number of non-homeostatic functions, including cell cycle regulation through the p38 MAPK pathway, and recent studies have indicated that it is required for Ca^2+ ^mediated growth arrest in pancreatic carcinoma cells. Some pancreatic cancers produce pathogenic amounts of parathyroid like hormones, however, which significantly increase Ca^2+ ^plasma concentrations and might be expected to block further cell growth. In this study we have investigated the expression and function of the p38 MAPK signaling pathway in Ca^2+ ^sensitive (T3M-4) and insensitive (FA6) pancreatic cancer cell lines. FA-6 cells, which are derived from a pancreatic adenocarcinoma that secretes a parathyroid hormone related peptide, exhibit only very low levels of p38 MAPK expression, relative to T3M-4 cells. Transfecting FA-6 cells with a p38 MAPK expression construct greatly increases their sensitivity to Ca^2+^. Furthermore, the reduction of p38 MAPK in T3M-4 cells significantly reduces the extent to which high levels of Ca^2+ ^inhibit proliferation. These results suggest that the low levels of p38 MAPK expression in FA-6 cells may serve to reduce their sensitivity to high concentrations of external Ca^2+ ^that would otherwise block proliferation.

## Background

The Calcium -Sensing Receptor (CaR) was cloned a decade ago, and has proven to be an important component in Ca^2+ ^homeostasis through its regulation of parathyroid hormone (PTH [1]). It also has a number of non-homeostatic functions including the control of ion channels and hormone secretion, and also in the regulation of cell cycle events [2]. Most notably, Ca^2+ ^stimulation of CaR promotes the proliferation of osteoblasts [3] and fibroblasts [4], together with a number of malignant cell types including myeloma and ovarian surface cancer [5], probably through activation of the EGF receptor [6]. Conversely, the growth of some cells is blocked, including colonic [7] and some pancreatic carcinomas [8]. In each case signaling through CaR is mediated by a specific Mitogen activated protein kinase (MAPK) pathway, characterized by and dependant upon p38 MAPK.

Hypercalcaemia due to the degradation of bone matrix by osteoclasts is commonly associated with a number of malignancies, most notably those of the breast [9], lung [10], testis and kidney [11], although it is also a feature of some pancreatic cancers [12]. The mechanism frequently underlying this process is the secretion of PTH-related peptide (PTHrP) by cancer cells [13]. This activates osteoblast cells that in turn promote osteoclasts to degrade the bone matrix, releasing large amounts of Ca^2+^. High Ca^2+^concentrations then stimulate further PTHrP secretion, resulting in increasingly severe bone loss and hypercalcaemia, and in many cases promotes further tumour growth [13,14].

In view of the generalized anti-proliferative effect of Ca^2+ ^on pancreatic adenocarcinoma cells (PACs), it is surprising that some PACs actually secrete high levels of PTHrP, thereby promoting hypercalcaemia. Here we compare two PAC derived cell lines, FA-6 [12] and T3M-4 [15], which have similar characteristic except that FA-6 secretes high levels of PTHrP [12]. Our results indicate that the relative insensitivity of FA-6 cells to Ca^2+ ^is mediated by a constitutively low level of p38 MAPK expression in these cells.

## Materials and methods

### Cell culture and treatment

FA-6 and T3M-4 cells were maintained in RPMI 1640 supplemented with 10% FBS, 1% P/S and 1% B16 granulocyte-macrophage colony stimulating factor (GM-CSF) conditioned media, and incubated at 37°C, 5% CO_2._

### P38 MAPK transfection

The full length reading frame of p38 MAPK (NM_002745) was amplified by PCR and cloned into the pCMV-script vector (Stratagene, USA) to give pCMVp38+. As a control, we also cloned the p38 MAPK reading frame lacking the ATG translation start site (pCMVp38). Transfections for both FA-6 and T3M-4 were performed in 35 mm plates with cells at 80% confluency. 2 μg of each vector were used per plate, together with 6 μl of GeneJammer transfection reagent (Stratagene, USA), with other conditions as described in the manufacturer's protocol.

### RNA extraction and RT-QPCR

Total RNA was extracted from FA-6 or T3M-4 cells using an RNeasy mini kit (Qiagen). 1 μg of RNA was used in subsequent reverse transcription reactions. This was mixed with a poly T15 oligo to 5 μg/ml and heated to 75°C for 5 minutes. After cooling on ice, the following additional reagents were added; dNTPs to 0.4 mM, RNase OUT (Promega) to 1.6 u/μl, Moloney Murine Leukemia Virus Reverse Transcriptase (M-MLRvT) RnaseH- point mutant (Promega) to 8 u/μl and the appropriate buffer (supplied by the manufacturer) to x1 concentration. The mixture was incubated for one hour at 37°C, heated to 70°C for two minutes and cooled on ice.

QPCR reactions were all performed in a total volume of 50 μl. For each we used 1 μl of the M-MLRvT reaction (as described above), 0.2 nmols of each primer and 25 μl of pre-mixed QPCR components (Sigma). All reactions were cycled at 94°C for 30 seconds, 55°C for 30 seconds and 72°C for 60 seconds, for 45 cycles.

QPCR was performed using the SYBR green labeling kit from Sigma. Thermal cycling and fluorescence detection was by a MX4000 (Stratagene Inc., USA). Semi-quantitative data was obtained by using measurements three cycles after reactions had risen above the base line, and were clearly in exponential increase. β-actin was used as a control for RNA recovery and cDNA synthesis, and all values are presented as a ratio of target to β-actin signal.

### Detection of p38 MAPK protein by western blotting

Whole cell lystaes were prepared from FA-6 and T3M-4 cells and 15 μg was used for each detection. Blotting was performed using the Western Breeze Chemuliminescent kit (anti-rabbit, Invitrogen, UK), according to the manufactures instructions. Primary detection was by a rabbit anti-p38 MAPK antibody (ab7952, AbCam, UK), at a final concentration of 0.1 μg/ml. Blots were stripped and re-probed with rabbit anti-beta actin (Abcam, UK, ab8227) to act as a loading control.

### siRNA silencing of p38 MAPK in T3M-4 cells

Cultured T3M-4 cells were plated in triplicate the day before the transfection procedure was started. Pre-validated siRNA (Ambion, id # 1634) was transfected at a final concentration of 100 nM. RNA was extracted from the cells 48 hours after transfection. mRNA levels were measured by real-time RT-PCR, as described above. As a positive control, we also transfected with siRNA to target GAPDH, and with a non-targeting siRNA, to act as a negative control. Both of these siRNAs are supplied in the Ambion 'siRNA starter kit', which was used for all of the transfections, using the manufacturer's protocol.

## Results and discussion

In order to compare the expression of CaR and its downstream signaling components in PTHrP-expressing and non-expressing PAC lines, we selected FA-6 [12] and T3M-4 [15]. The former was established from a biopsied lymph node removed from a patient with PAC associated with humoral hypercalcaemia of malignancy, and secretes both PTHrP together with a TNFα-type bone reabsorbing activity. Likewise, T3M-4 was also established from a biopsied lymph node taken from a PAC patient, but unlike FA-6, T3M-4 is not known to secrete a bone-reabsorbing activity.

**Figure 1:**
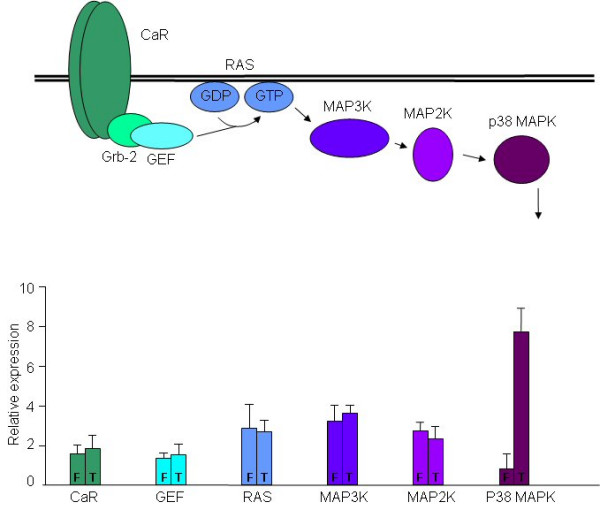
The relative expression of components in the CaR/p38 MAPK signaling cascade, as determined by QPCR in FA-6 (F) and T3M-4 (T) pancreatic carcinoma cell lines. The relative level of expression of each component is shown below a schematic pathway for this signaling cascade. Each value shown is a mean of six independent experiments; error bars represent the standard error of the mean.

**Figure 2:**
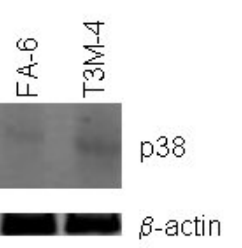
Western blot detection of p38 MAPK protein in FA-6 and T3M-4 cells. Beta-actin is included as a loading control.

In order to assess the transcription of CaR and its downstream signaling components, we extracted RNA from T3M-4 and FA-6 cells in culture. The relative amounts of specific transcripts in this pathway were then determined by quantitative PCR (QPCR). In brief, this pathway consists of CaR itself linked via an adaptor protein (Grb-2) to a guanine exchange factor (GEF), which upon Ca^2+ ^signaling catalyses the exchange of GTP for GDP by membrane bound Ras protein. GTP-bound Ras then activates MAP3K that in turn phosphorylates and thereby activates MAP2K, and this then activates p38 MAPK in the same manner (Fig 1; reviewed in ref [16]). The QPCR results indicate that whilst the majority of the CaR signaling pathway components, including CaR itself, are expressed at essentially the same level in the two different cell lines, p38 MAPK is specifically downregulated in FA-6 cells. The difference in expression levels is approximately 8 fold. This difference is also reflected at the protein level, as western blotting whole cell protein extracts from both cell lines reveals a significantly lower expression of p38 MAPK protein in FA-6 cells (Fig 2).

**Figure 3:**
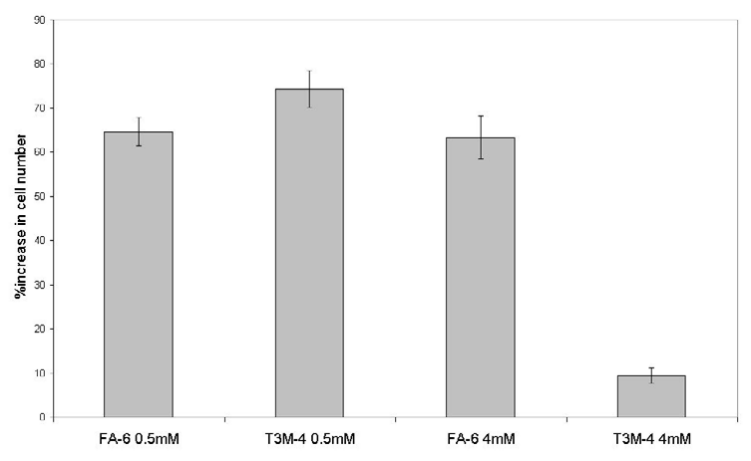
FA-6 cells, but not T3M-4 cells continue to proliferate when exposed to high concentrations (4 mM) of Ca^2+^. Results are the mean of three experiments, error bars show the SEM.

**Figure 4:**
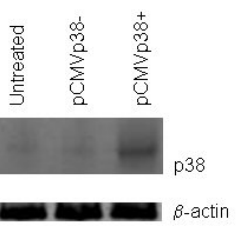
Transfection of FA-6 cells with p38 MAPK. FA-6 cells were tansfected with a full length clone of p38 MAPK (pCMVp38+), or with an almost identical clone lacking a translation start site (pCMVp38-). The levels of p38 MAPK protein were subsequently analyzed by western blotting using lysates of treated cells and an anti- p38 MAPK antibody. Loading control: β-actin.

**Figure 5:**
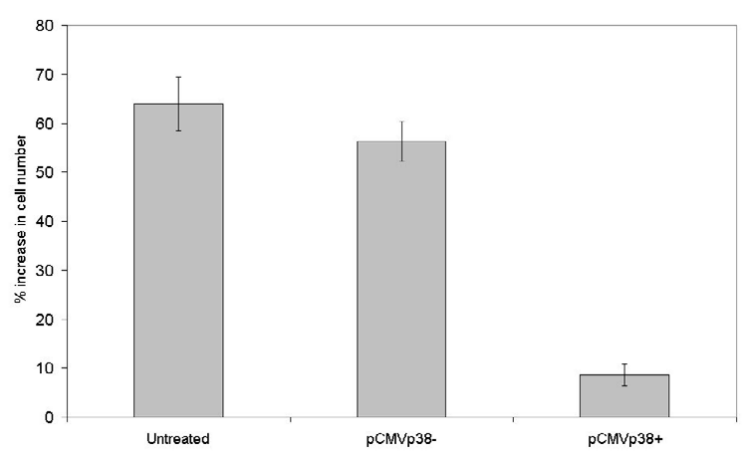
Forced expression of p38 MAPK sensitizes FA-6 cells to high Ca^2+ ^concentrations. FA-6 cells were transfected with pCMVp38+ or pCMVp38- and cultured in high Ca^2+^. The percentage increase in cell number over 24 hours is shown for the mean of three experiments, error bars show the SEM.

The relatively low level of p38 MAPK expression in FA-6 cells suggests that FA-6 cells could have a different Ca^2+ ^sensitivity to T3M-4 cells. In order to test this, we measured the proliferation of cells in the presence of low (0.5 mM) and high (4 mM) Ca^2+ ^(Fig 3). Although the rate of proliferation is similar for both cell types at the lower concentration, a higher concentration of Ca^2+ ^causes a significant reduction in T3M-4 proliferation but not of FA-6 proliferation. To test whether the relatively low expression of p38MAPK could be responsible for this difference, we transfected FA-6 cells with a vector containing the full length reading frame of p38MAPK (pCMVp38+), or with one containing the same insert but without the ATG start codon (pCMVp38-) to act as a control. FA-6 cells transfected with pCMVp38+ showed significantly raised levels of p38MAPK protein (Fig 4), and also a considerable decrease in proliferation when in the presence of 4 mM Ca^2+ ^(Fig 5), whilst cells FA-6 cells transfected with pCMVp38- did not.

**Figure 6:**
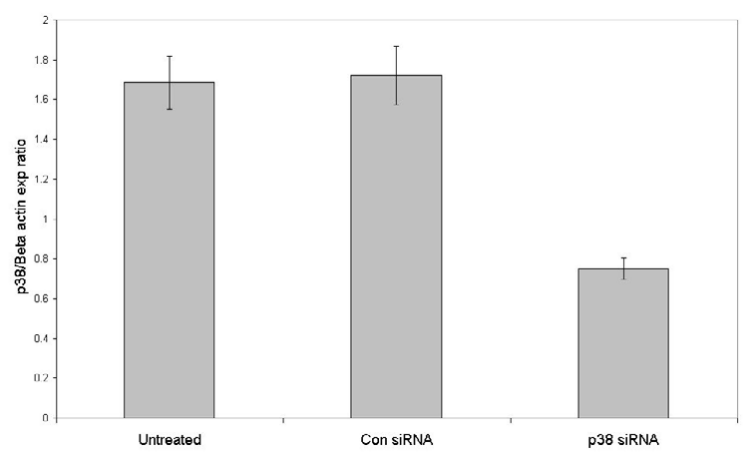
siRNA-mediated depletion of p38 MAPK RNA in T3M-4 cells. The relative expression of p38 MAPK is shown as a proportion of β-actin. Cells were either left untreated or were transfected with control siRNA or siRNA targeting p38 MAPK. The results shown are the mean of three experiments, error bars represent the SEM.

**Figure 7:**
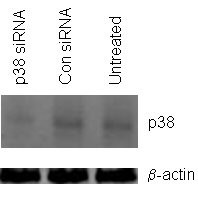
Western blot detection of p38 MAPK protein in untreated, control siRNA and p38 MAPK siRNA transfected T3M-4 cells. β-actin is included as a loading control.

**Figure 8:**
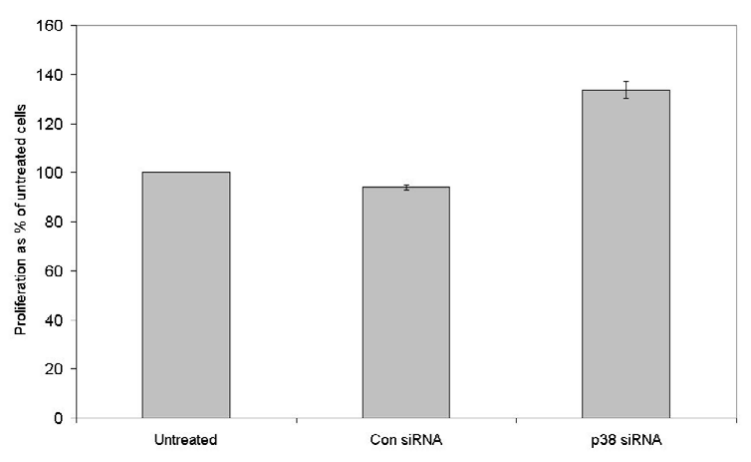
Cells transfected with siRNA targeting p38 MAPK show significantly increased proliferation in the presence of 4 mM Ca^2+^. T3M-4 cells were transfected with control or p38 MAPK siRNA, as shown and were cultured in medium supplemented with 4 mM Ca^2+^. The proliferation of each population is shown relative to that of untreated cells over 48 hours.

If a reduced level of p38 MAPK expression is responsible for blocking the response to Ca^2+ ^in FA-6 cells, then it is possible that reducing the relatively high levels of p38 MAPK in T3M-4 cells could also reduce their sensitivity to Ca^2+^. To address this, we targeted p38 MAPK in T3M-4 cells using a sequence specific short interfering RNA (siRNA). This resulted in a substantial (72%) reduction in p38 MAPK RNA, as measured by RT-QPCR (Fig 6), and a corresponding reduction in p38 MAPK protein (Fig 7). T3M-4 cells treated in this way showed a reduced sensitivity to Ca^2+ ^mediated inhibition of proliferation, whilst untreated cells and those treated with a non-targeting siRNA did not (Fig 8).

The analysis of differential transcription between cells and tissues is becoming increasingly important in diagnosis and prognosis, and also in understanding the physiology of different cell types. Studies using micro array based approaches have indicated that changes in the transcription of relatively small groups of genes may underlie the difference between normal and malignant states [17], and that these genes can be subdivide into functional distinct subclasses, for example those involved in proliferation and growth. In this study, we show that a single, stable transcriptional change between different pancreatic carcinoma cell lines may influence their response to distinct environmental signals, in this case the extracellular concentration of Ca^2+^. This change is in the transcription of p38 MAPK, a key component of the CaR-mediated proliferative/anti-proliferative affect of Ca^2+ ^[18]. It is intriguing that p38 MAPK should be so strongly downregulated in FA-6 cells, which are derived from a tumour that both promotes hypercalcaemia through PTHrP secretion whilst escaping its anti-proliferative effects seen in other PACs. Greatly reduced p38 MAPK expression is thus an example of a specific change in the CaR signaling pathway that can block its activity, and of a selection for a specific transcriptomic change that allows an escape from anti-proliferative signals.

## Competing interests

The author(s) declare that they have no competing interests.

## Authors' contributions

All authors participated in the design of experiments. BF was responsible for qRT-PCR studies. RM performed transfections and the siRNA experiments and drafted the manuscript. HP assisted with data analysis and interpretation. All authors read and approved the final manuscript.
